# Supplementary material for: Classification of antimicrobial mechanism of action using dynamic bacterial morphology imaging
Source: Sci Rep. 2022 Jul 1;12:11162. doi: 10.1038/s41598-022-15405-1 (PMC9249789; doi:10.1038/s41598-022-15405-1)
Supplement: Supplementary file 7 — Supplementary Information. [file 41598_2022_15405_MOESM7_ESM.pdf]

Supplemental Material

Title: Classification of antimicrobial mechanism of action using dynamic bacterial morphology imaging

Xudong Ouyang, Jelmer Hoeksma, Ronnie J.M. Lubbers, Tjalling K. Siersma, Leendert W. Hamoen and Jeroen den Hertog

Legends to supplemental movies S1-S6

Fig S1. B. subtilis growth curves in the presence of 1.0x, 2.5x or 5.0x MIC.

Fig. S2. Additional imaging profiles.

Table S1. List of antimicrobials used in this study

Legends to supplemental movies S1-S6

Dynamic profiling patterns of cells upon antimicrobial treatment. *B. subtilis* cells were stained with FM4-64 (red, cell membrane) and SYTO-9 (green, nucleoid) and imaged by time lapse confocal fluorescence microscopy for 60 min with 3 min intervals. Cells were treated with 1% DMSO (control, Movie S1) or antimicrobials (2.5 × MIC): ampicillin, Movie S2; CCCP, Movie S3; chloramphenicol, Movie S4; moxifloxacin, Movie S5; Rifampin, Movie S6. Antimicrobials were added after 6 min; time is indicated in the top left corner as hh:mm:ss. Representative cells are shown.

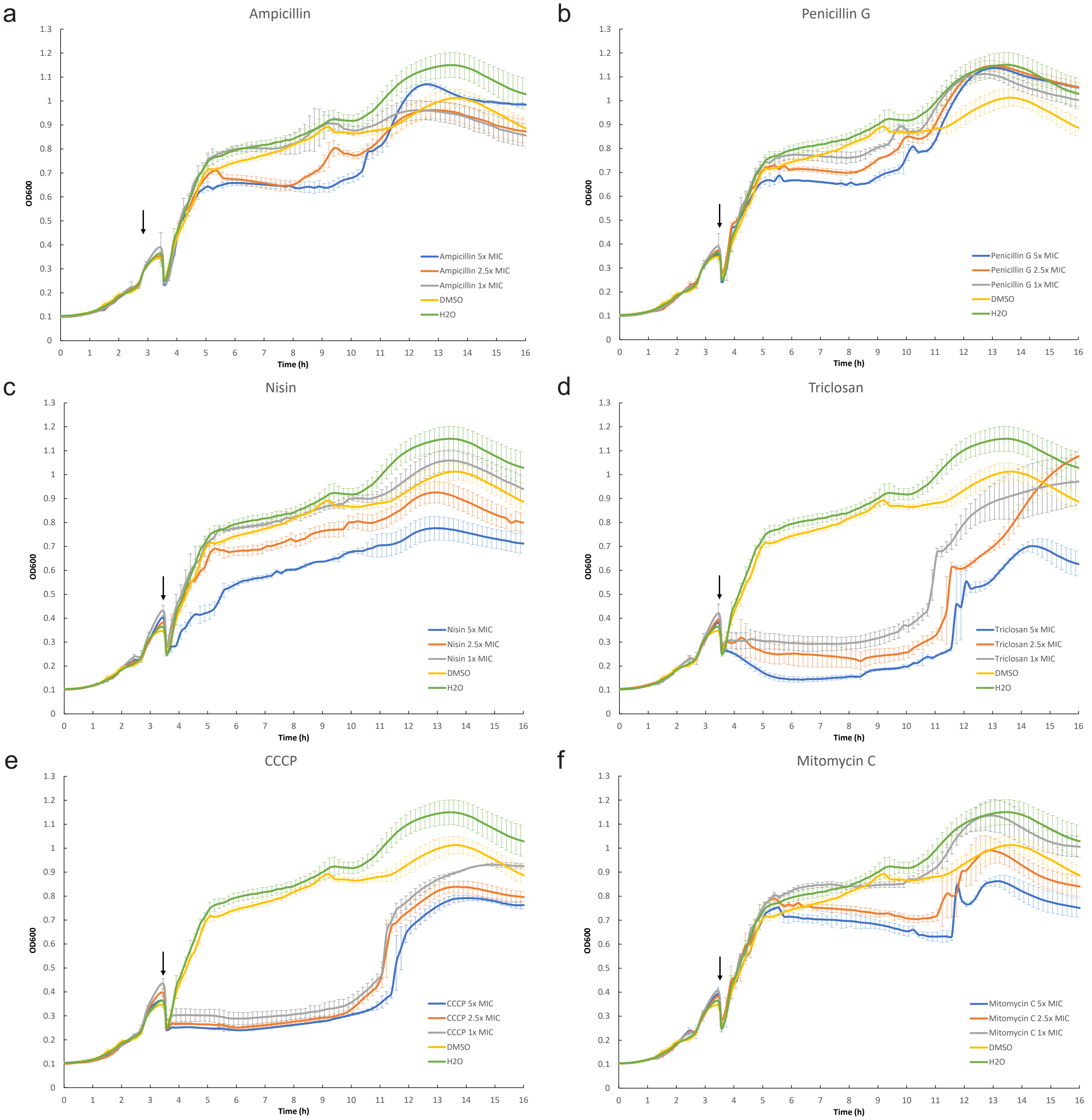

Fig S1. ***B. subtilis*** **growth curves in the presence of 1.0x, 2.5x or 5.0x MIC.** (a) Ampicillin, (b) penicillin G, (c) nisin, (d) triclosan, (e) CCCP, (f) mitomycin C, (g) chloramphenicol, (h) moxifloxacin, (i) actinomycin, (j) fusidic acid, (k) gentamcyin, (l) nalidixic acid, (m) rifampin and (n) vancomycin. *B. subtilis* strain 168 was grown overnight in LB medium at 37°C, 200 rpm. Overnight culture was diluted in LB, distributed in 96-wells plates and incubated at 37°C till an OD600 of approximately 0.3-0.4 was reached. Antibiotics with a concentration corresponding to 1x (grey), 2.5x (orange) and 5x (blue) MIC with final concentration DMSO of 1% was added (marked with a black arrow), mixed by pipetting and incubated at 37°C. As controls, a solvent control with final concentration of 1% DMSO (yellow) and a growth control with H_2O (green) were added to the experiment. Error bars represent the standard deviation of triplicates. OD600 was determined every 15 min. Note that administration of antibiotic caused a minor disruption. In most cases, the response to 1.0 x MIC and 2.5 x MIC was very similar in the first 60 min. After 10 hr treatment, the response to the varying concentrations of antibiotics diverged and major differences in OD600 were observed.

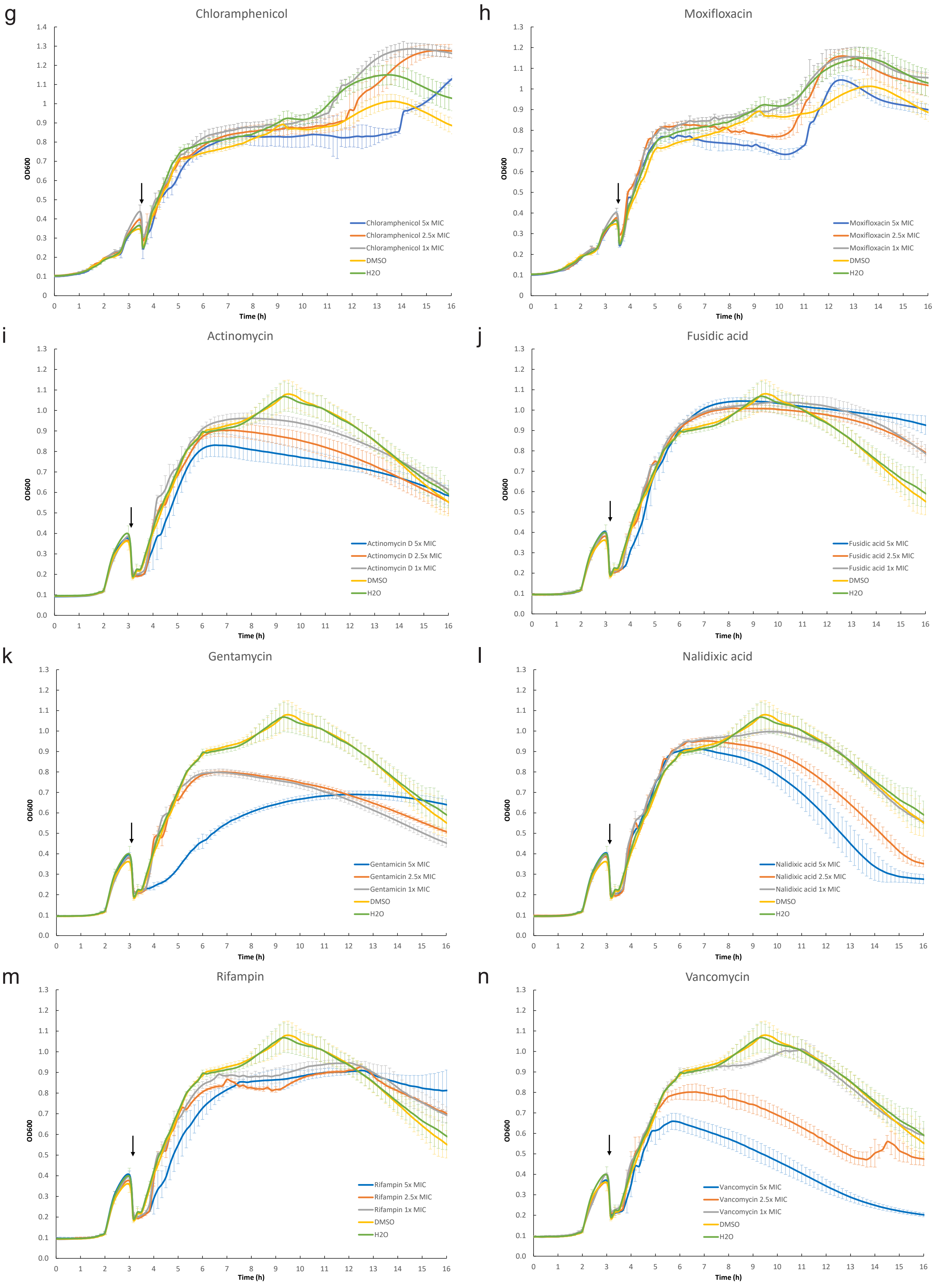

Fig S1. ***B. subtilis*** **growth curves in the presence of 1.0x, 2.5x or 5.0x MIC (continued).**

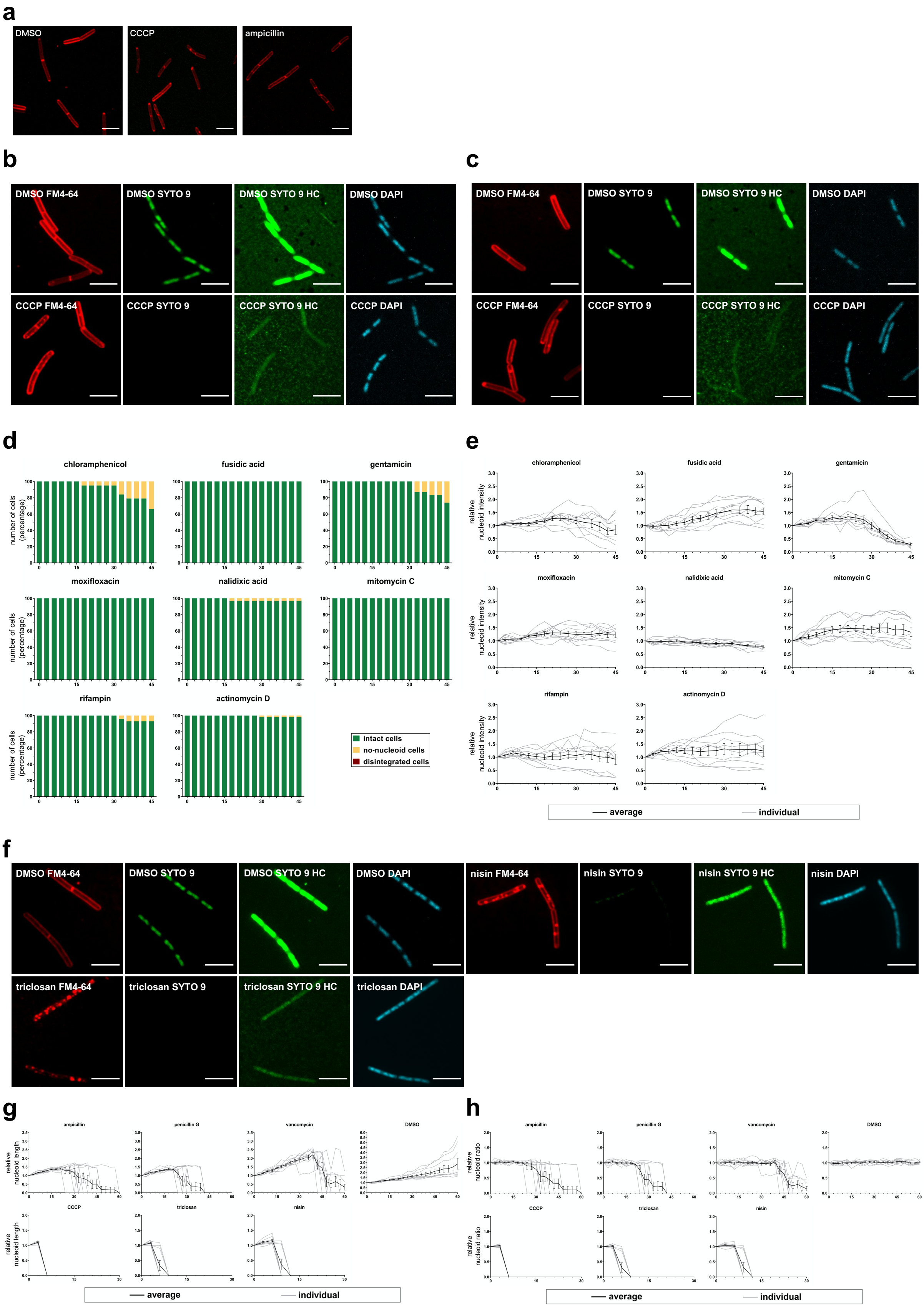
a
DMSO
CCCP
ampicillin
b
DMSO FM4-64
DMSO SYTO 9
DMSO SYTO 9 HC
DMSO DAPI
CCCP FM4-64
CCCP SYTO 9
CCCP SYTO 9 HC
CCCP DAPI
c
DMSO FM4-64
DMSO SYTO 9
DMSO SYTO 9 HC
DMSO DAPI
CCCP FM4-64
CCCP SYTO 9
CCCP SYTO 9 HC
CCCP DAPI
d
chloramphenicol
fusidic acid
gentamicin
moxifloxacin
nalidixic acid
mitomycin C
rifampin
actinomycin D
number of cells (percentage)
intact cells
no-nucleoid cells
disintegrated cells
e
chloramphenicol
fusidic acid
gentamicin
moxifloxacin
nalidixic acid
mitomycin C
rifampin
actinomycin D
relative nucleoid intensity
average
individual
f
DMSO FM4-64
DMSO SYTO 9
DMSO SYTO 9 HC
DMSO DAPI
nisin FM4-64
nisin SYTO 9
nisin SYTO 9 HC
nisin DAPI
triclosan FM4-64
triclosan SYTO 9
triclosan SYTO 9 HC
triclosan DAPI
g
ampicillin
penicillin G
vancomycin
DMSO
CCCP
triclosan
nisin
relative nucleoid length
average
individual
h
ampicillin
penicillin G
vancomycin
DMSO
CCCP
triclosan
nisin
relative nucleoid ratio
average
individual

Fig. S2. Additional imaging profiles.

(a) Cell permeability assay. *B. subtilis* cells were treated with antimicrobials (2.5 × MIC) or 1% DMSO (control) for 60 min. Cells were stained with FM4-64 (red, cell membrane) and SYTOX-Green (green, nucleoid), and imaged by confocal fluorescence microscopy. Representative images are shown. Scale bar is 5 μm.

(b) Dual nucleoid staining of cells following CCCP treatment for 3 min. *B. subtilis* cells were treated with CCCP (2.5 × MIC) or 1% DMSO (control) for 3 min. Cells were stained with FM4-64 (red, cell membrane), SYTO-9 (green, nucleoid) and DAPI (blue, nucleoid), immobilized and imaged by confocal fluorescence microscopy. Representative images are shown. Scale bar is 5 μm. SYTO-9 graphs were shown both in normal contrast and high contrast (HC).

(c) Dual nucleoid staining of cells following CCCP treatment for 60 min. *B. subtilis* cells were treated with CCCP (2.5 × MIC) or 1% DMSO (control) for 60 min. Cells were stained with FM4-64 (red, cell membrane), SYTO-9 (green, nucleoid) and DAPI (blue, nucleoid), and imaged by confocal fluorescence microscopy. Representative images are shown. Scale bar is 5 μm. SYTO-9 graphs were shown both in normal contrast and high contrast (HC).

(d) Changes in cell status upon antimicrobial treatment. *B. subtilis* cells were treated with antimicrobials (2.5 × MIC) as indicated. Cells were stained with FM4-64 (red, cell membrane) and SYTO-9 (green, nucleoid), and imaged by time lapse confocal fluorescence microscopy with 3 min intervals. Three distinct cell types were identified: intact cells (cells with visible membrane and nucleoid fluorescence), no-nucleoid cells (cells with apparently intact membrane, but without visible nucleoid florescence) and disintegrated cells (cells with disintegrated membrane and no detectable nucleoid). The number of cells of the three types were counted from biological triplicate imaging series (n > 20). The ratio of cell types, intact cells (green), no-nucleoid cells (yellow) and disintegrated cells (red), was determined and plotted as percentage (y-axis, numbers in %) over time (x-axis, min). See Fig. 4a for DMSO control as this figure is an extension.

(e) Changes in nucleoid intensity upon antimicrobial treatment. *B. subtilis* cells were treated with antimicrobials (2.5 × MIC) as indicated. Cells were stained and imaged as in (d). Cell morphology data were collected from 9 cells in total per treatment, i.e. technical triplicates from biological triplicate imaging series. Overall nucleoid intensity, i.e. the SYTO-9 fluorescence intensity inside a whole cell, corrected for background, was determined for individual cells over time and was depicted as percentage of the value at the start (t = 0min) (y-axis) over time (x-axis, min). The mean of antimicrobial-treated cells was plotted in black

with error bars representing the SEM. Gray lines represent individual antimicrobial-treated cells on which the mean was based. See Fig. 4b for DMSO control as this figure is an extension.

(f) Dual nucleoid staining of cells following treatment with antimicrobials from cell membrane class or harzianic acid (HA) for 3 min. *B. subtilis* cells were treated with antimicrobials (2.5 × MIC) as indicated or 1% DMSO (control) for 3 min. Cells were stained with FM4-64 (red, cell membrane), SYTO-9 (green, nucleoid) and DAPI (blue, nucleoid), and imaged by confocal fluorescence microscopy. Representative images are shown. Scale bar is 5 µm. SYTO-9 graphs were shown both in normal contrast and high contrast (HC).

(g) Changes in nucleoid length upon antimicrobial treatment. *B. subtilis* cells were treated with antimicrobials (2.5 × MIC) as indicated or 1% DMSO (control). Cells were stained, imaged and analyzed as in (d). Nucleoid length, the addition of the length of all the nucleoids inside one cell, was determined for individual cells and depicted as percentage of the value at the start (t = 0min) (y-axis) over time (x-axis, min). The mean of antimicrobial-treated cells was plotted in black with error bars representing the SEM. Gray lines represent individual antimicrobial-treated cells on which the mean was based. This figure is an extension of Fig. 5a, where the first 45 min of DMSO control has already shown.

(h) Changes in the ratio of nucleoid length to the cell length upon antimicrobial treatment. *B. subtilis* cells were treated with antimicrobials (2.5 × MIC) as indicated or 1% DMSO (control). Cells were stained, imaged and analyzed as in (d). The ratio of the nucleoid length to the cell length, was calculated for individual cells over time and was depicted as percentage of the value at the start (t = 0min) (y-axis) over time (x-axis, min). The mean of antimicrobial-treated cells was plotted in black with error bars representing the SEM. Gray lines represent individual antimicrobial-treated cells on which the mean was based.

Table S1. List of antimicrobials used in this study

Class	Sub-class	Antimicrobials	MIC* (mg/L)	Mode of Action
Cell wall	Penicillin	Ampicillin	5	Binding to Penicillin-binding protein[1]
	Penicillin	Penicillin G	5	Binding to Penicillin-binding protein[1]
	Glycopeptide	Vancomycin	0.25	Binding to D-Ala-D-Ala moiety of peptidoglycan[2]
Cell membrane	Proton ionophore	CCCP	0.4	Transporting protons across membranes[3]
	Polychloro phenoxy phenols	Triclosan	2.5	Disrupting cellular ionic homeostasis at bactericidal concentration[4]
	Lantibiotics	Nisin	2.5	Generating pores on cell membrane[5]
Protein	Amphenicols	Chloramphenicol	2.5	Inhibiting the peptidyl transferase activity[6]
	Fusidanes	Fusidic acid	0.13	Binding to the elongation factor G (EF-G)[7]
	Aminoglycoside	Gentamicin	2.5	Binding to the A-site on the 16S ribosomal RNA[8]
DNA	Quinolone	Moxifloxacin	0.05	Inhibiting DNA gyrase A and topoisomerase IV[9]
	Quinolone	Nalidixic acid	1.5	Inhibiting DNA gyrase A and topoisomerase IV[10]
	Mitomycin	Mitomycin C	0.06	Cross-linking and alkylating of DNA[11]
RNA	Rifamycin	Rifampin	0.13	Inhibiting bacterial RNA polymerase[12]
	Actinomycines	Actinomycin D	0.03	Inhibiting bacterial RNA synthesis by binding to DNA at the transcription initiation complex[13]

* Determined by our stain of *Bacillus subtilis* strain 168 in this study.

References

1. Williamson, R., Hakenbeck, R. & Tomasz, A. In vivo interaction of β-lactam antibiotics with the penicillin-binding proteins of Streptococcus pneumoniae. *Antimicrob. Agents Chemother.* **18**, 629–637 (1980).

2. Boger, D. L. Vancomycin, teicoplanin, and ramoplanin: Synthetic and mechanistic studies. *Med. Res. Rev.* **21**, 356–381 (2001).

3. Kasianowicz, J., Benz, R. & McLaughlin, S. The kinetic mechanism by which CCCP (carbonyl cyanide m-Chlorophenylhydrazone) transports protons across membranes. *J. Membr. Biol.* **82**, 179–190 (1984).

4. Russell, A. D. Whither triclosan? *J. Antimicrob. Chemother.* **53**, 693–695 (2004).

5. Prince, A. *et al.* Lipid-II Independent Antimicrobial Mechanism of Nisin Depends on Its Crowding and Degree of Oligomerization. *Sci. Rep.* **6**, 1–15 (2016).

6. Siibak, T. *et al.* Erythromycin- and chloramphenicol-induced ribosomal assembly defects are secondary effects of protein synthesis inhibition. *Antimicrob. Agents Chemother.* **53**, 563–571 (2009).

7. Dobie, D. & Gray, J. Fusidic acid resistance in Staphylococcus aureus. *Arch. Dis. Child.* **89**, 74–77 (2004).

8. Borovinskaya, M. A. *et al.* Structural basis for aminoglycoside inhibition of bacterial ribosome recycling. *Nat. Struct. Mol. Biol.* **14**, 727–732 (2007).

9. Drlica, K. & Zhao, X. DNA gyrase, topoisomerase IV, and the 4-quinolones. *Microbiol. Mol. Biol. Rev.* **61**, 377–392 (1997).

10. Sugino, A., Peebles, C. L., Kreuzer, K. N. & Cozzarelli, N. R. Mechanism of action of nalidixic acid: Purification of Escherichia coli nalA gene product and its relationship to DNA gyrase and a novel nicking-closing enzyme. *Proc. Natl. Acad. Sci. U. S. A.* **74**, 4767–4771 (1977).

11. Paz, M. M., Zhang, X., Lu, J. & Holmgren, A. A new mechanism of action for the anticancer drug Mitomycin C: Mechanism-based inhibition of thioredoxin reductase. *Chem. Res. Toxicol.* **25**, 1502–1511 (2012).

12. Wehrli, W. Rifampin: Mechanisms of action and resistance. *Rev. Infect. Dis.* **5**, S407–S411 (1983).

13. Sobell, H. M. Actinomycin and DNA transcription. *Proc. Natl. Acad. Sci. U. S. A.* **82**, 5328–5331 (1985).
